# Five-Membered Heterocyclic Sulfonamides as Carbonic Anhydrase Inhibitors

**DOI:** 10.3390/molecules28073220

**Published:** 2023-04-04

**Authors:** Andrea Angeli, Niccolò Paoletti, Claudiu T. Supuran

**Affiliations:** NEUROFARBA Department, Sezione di Scienze Farmaceutiche, University of Florence, Via Ugo Schiff 6, Sesto Fiorentino, 50019 Florence, Italy; andrea.angeli@unifi.it (A.A.); niccolo.paoletti@unifi.it (N.P.)

**Keywords:** carbonic anhydrase, carbonic anhydrase inhibitors, heterocyclic sulfonamides, five-membered heterocycle, metalloenzyme

## Abstract

The development of heterocyclic derivatives has progressed considerably over the past decades, and many new carbonic anhydrase inhibitors (CAIs) fall into this field. In particular, five-membered heterocyclic sulfonamides have been generally shown to be more effective inhibitors compared to six-membered rings ones. Despite the importance of oxygen and nitrogen five-membered heterocyclic aromatic rings in medicinal chemistry, the installation of sulfonamide moiety on such heterocycles has not received much attention. On the other hand, 1,3,4-thiadiazole/thiadiazoline ring-bearing sulfonamides are the scaffolds which have been widely used in a variety of pharmaceutically important CAIs such as acetazolamide, metazolamide and their many derivatives obtained by using the tail approach. Here, we reviewed the field focusing on the diverse biological activities of these CAIs, such as antiglaucoma, antiepileptic, antitumor and antiinfective properties. This review highlights developments involving five-membered heterocyclic sulfonamides over the last years, with a focus on their pharmacological/clinical applications.

## 1. Introduction

**Scheme 1:**
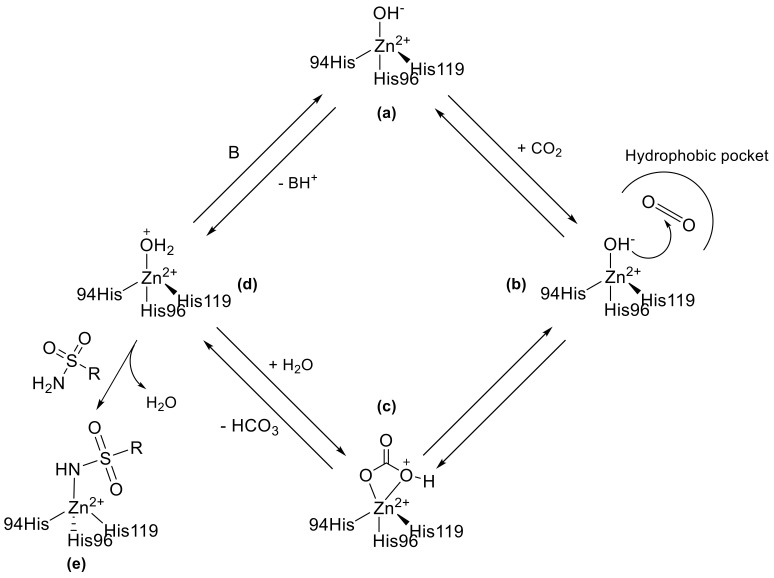
The CO_2_ hydration reaction mechanism of Zn-CA II and inhibition with sulfonamide binders.

Carbonic anhydrases (CAs, E.C. 4.2.1.1) are a family of metalloenzymes that catalyse the reversible hydration of carbon dioxide to bicarbonate and protons following a two-step catalytic mechanism [1,2,3,4,5]. The initial phase involves a nucleophilic attack of CO_2_ to a Zn^2+^-bound hydroxide ion resulting in the formation of HCO_3_^−^. This is then replaced at the active site by a water molecule (Scheme 1). The second step, which is rate limiting, regenerates the catalytically active Zn^2+^-bound hydroxide ion through a proton transfer reaction from the Zn^2+^-bound water molecule to an exogenous proton acceptor or to an active site residue, as shown in Scheme 1 [1,2,3,4,5]. These enzymes are widely distributed in nature and, nowadays, are classified in eight different genetic unrelated families, namely the α-, β-, γ-, δ-, η-, ζ-, θ and –ι classes [6,7,8,9,10]. This simple reaction plays important roles in various physiological processes, including acid-base balance, regulation of intra-/extracellular pH, metabolism and carbon dioxide transport [11,12,13,14]. In humans, fifteen different isoforms were found that fall in the α-class each with distinct subcellular localization, tissue distribution, and physiological functions. Twelve isoforms (CA I−IV, VA−VB, VI−VII, IX, and XII−XIV) show a variable degree of enzymatic activity, whereas three of them (CA VIII, X, and XI), the so-called CA-related proteins (CARPs), are devoid of CO_2_ hydrase catalytic activity [15]. For these reasons, the different CA isoforms have emerged as attractive targets for therapeutic intervention and many efforts were made in the design of novel and effective carbonic anhydrase inhibitors (CAIs). To date, CA inhibition has been shown to have therapeutic potential in the treatment of a wide range of diseases, including edema, glaucoma, epilepsy, obesity, neuropathic pain, and cancer [16,17,18,19,20].

In particular, five-membered heterocyclic sulfonamides are a class of compounds that have been extensively studied due to their high potency and selectivity inhibition against CA isoforms. The study of this type of scaffolds first gained attention in 1945 due to the trailblazing research conducted by Davenport, who discovered the thiophene-2-sulfonamide was more effective as CAI compared to six-member aromatic/heterocyclic scaffold, including sulfanilamide [21]. Building upon this initial discovery, a significant number of sulfonamides containing various heterocyclic rings such as imidazoles, benzimidazoles, benzothiazole, thiazole, pyrazine, and others were synthesized and evaluated for their ability to inhibit hCAs [22,23]. These investigations resulted in two key observations: (i) sulfonamides bearing five-membered rings tended to be more potent inhibitors than those with six-membered rings; (ii) the presence of nitrogen and sulfur atoms within the ring was linked to improved inhibitory activity [15].

In this review, we will discuss the current state of research on five-membered heterocyclic sulphonamides acting as CAIs, providing an overview of the current understanding of five-membered heterocyclic sulfonamides and their potential in therapy.

## 2. Five-Membered Heterocyclic Sulfonamides

### 2.1. Oxygen and Nitrogen Containing Five-Membered Heterocyclic Sulfonamides

**Scheme 2:**
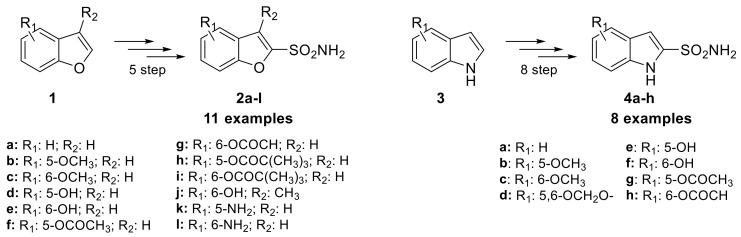
Schematic synthesis of benzofuran **2a**–**l** and indole sulfonamides **4a**–**h** by Graham et al.

Despite the importance of oxygen and nitrogen five-membered heterocyclic aromatic ring in medicinal chemistry, the installation of sulfonamide moiety on such simple rings for obtaining CAIs has not received much attention. More than 30 years ago, Graham and co-workers investigated the potential of heterocyclic furan-bearing sulfonamides as CAIs, studying their use as topically applied ocular hypotensive agents in the treatment of glaucoma [24]. In their research, they focused on the structural class of benzofuran-2-sulfonamides, synthesizing 11 examples through a five-step process starting from the benzofuran scaffold (**1**) and incorporated various functional groups (**2a**–**l**) such as amines, hydroxyls, and methoxys (Scheme 2).

**Scheme 3:**
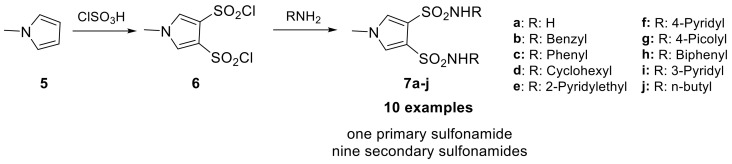
Schematic synthesis of *N*-methyl pyrrole disulfonamides by Abas et al.

However, the electrophilic proprieties of this class of compounds had a propensity to cause ocular and dermal sensitization. This was evaluated through their reaction rate with reduced glutathione (GSH), which was found to show a good correlation with dermal sensitization potential [25]. Of the 11 benzofuran sulfonamide examples synthesized, two were discovered to be moderate sensitizers in the Magnusson–Kligman dermal sensitization potential assay. Similarly, the isosteric indole sulfonamides were synthesized from five different indole scaffolds (**3**) in 8 steps, leading to eight examples (**4a**–**h**). Only one of these, the 5-hydroxyindolesulfonamide, was tested in the Magnusson-Kligman dermal sensitization assay and was found to be a strong sensitizer [25]. These results likely contributed to the limited investigations of these compounds over the last 30 years and a lack of interest in other of their congeners. In 2021, Abas et al. reported sulfonamides bearing pyrrole heterocycles, synthesizing 10 examples and evaluating their inhibition constant against an unspecified CA isoform [26]. These compounds were synthesized from the *N*-methyl pyrrole scaffold (**5**) using chlorosulfonic acid, yielding *N*-methyl pyrrole disulfonyl chloride derivative (**6**), which were then reacted with different amines to produce secondary or primary sulfonamide compounds **7a**–**j** (Scheme 3).

All these compounds were evaluated in vitro against MCF-7 cancer cells, displaying good cytotoxicity, and some of them showed selective toxicity towards MCF-7 cells compared to non-cancerous HaCaT. This selective activity was attributed to inhibition of tumor-associated isoforms CA IX and XII, which are overexpressed in MCF-7 cells and play a role in complex processes associated with tumorigenesis.

**Scheme 4:**
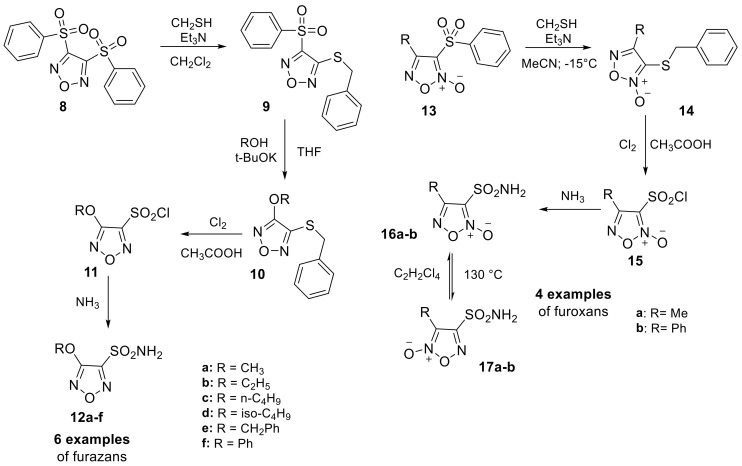
Schematic synthesis of furazan (**12a**–**f**) and (furoxan **16a**–**b** and **17a**–**b**) sulfonamides by Gasco and Fruttero group.

The shift from simple oxygen or nitrogen five-membered heterocyclic sulfonamides to more complex heteroaromatic scaffolds containing both atoms was provided by Gasco and Fruttero group in 2014. This led to the discovery of new heteroaromatic sulfonamides, such as 1,2,5-oxadiazole (furazan) sulfonamide derivatives (**12a**–**f**) or 2-oxides (furoxans) (**16a**–**b** and **17a**–**b**) [27]. The authors described a synthetic method to obtain these two scaffolds, although in this review, we will only focus on compounds that directly bear the sulfonamide moiety, in a multi-step procedure previously reported in one of their publications (Scheme 4) [28].

All six examples of furazan and four of furoxan were evaluated against four different human (h) hCAs including the widely expressed hCA I, II and the two isoforms that are predominantly associated and overexpressed in many tumors, hCA IX and XII. These derivatives displayed high inhibitory activity against all the isoforms considered in their study, demonstrating the possibility of modulating this activity through structural modification. In a subsequent study, the authors evaluated the ability of these compounds to decrease the IOP in vivo and discovered that they had stronger IOP lowering activity than dorzolamide [27].

**Scheme 5:**
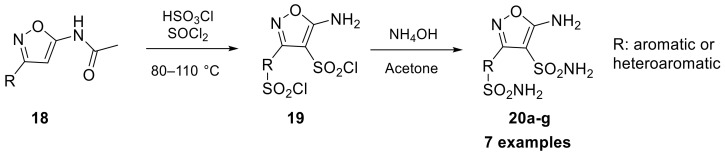
Schematic synthesis of isoxazole sulfonamides **20a**–**g** developed by Supuran’s group.

In 2017, isoxazole scaffold-bearing sulfonamides were studied by the Supuran group, offering the opportunity to introduce two primary sulfonamides into the same molecule, starting from compound **18** as reported in Scheme 5 [29].

The inhibitory proprieties of these isoxazole sulfonamide compounds (**20a**–**g**) were assayed against four hCAs, including hCA I, II, IV, and VII. The results of these assessments, in terms of constant of inhibition, suggested that the primary sulfonamide group may not act as a zinc-binding group (ZBG), but as a component of the isoxazole periphery. However, the presence of a bis-sulfonamide group appears to restore inhibitory properties that were not seen in mono-sulfonamides, pointing to the possibility of an alternative zinc anchoring mode for bis-sulfonamides. This new mode of anchoring may offer benefits for selective targeting of hCA IV and VII [29]. 

**Figure 1:**
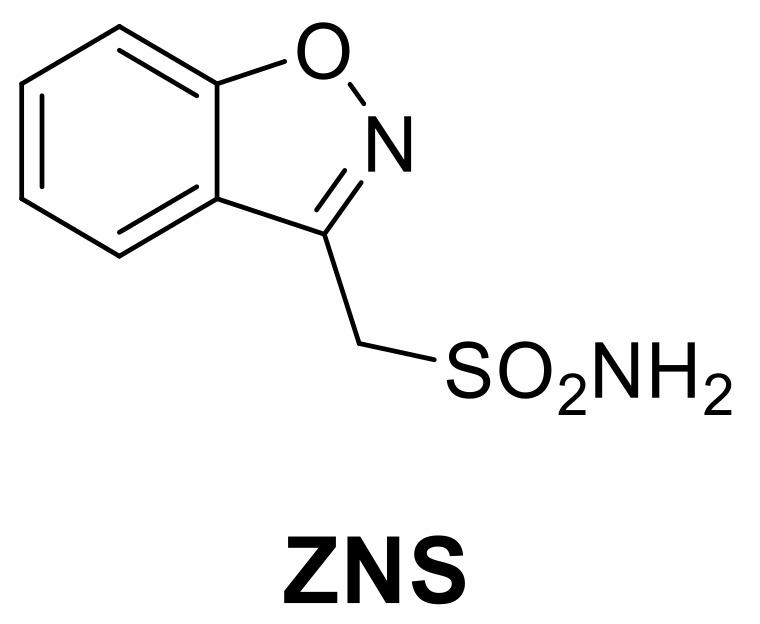
Structures of clinically approved Zonisamide (**ZNS**).

To conclude the discussion on heterocycles with nitrogen and oxygen, it is important to mention Zonisamide (**ZNS**), a benzisoxazole derivative (Figure 1) approved by the FDA in 1998 for the treatment of seizures associated with epilepsy and which shows significant CA inhibitory properties. [30].

**ZNS** is a second-generation antiepileptic drug with a complicated mechanism of action, which includes: (1) block of the neuronal voltage-dependent sodium channel [31], (2) block of the neuronal voltage-dependent T-type calcium channels [32], and (3) enhancement of GABAergic transmission [33,34]. Furthermore, in 2005, the Supuran group demonstrated its inhibition of hCA II (K_i_ 35 nM) and hCA VA (K_i_ 20 nM) highlighting the participation of CA inhibitory activity in its mechanism of action and, at the same time, explaining the weight loss of patients treated with **ZNS** [35]. In fact, the inhibition of mitochondrial hCA VA/VB isoforms leads to a reduction in body weight through the interaction with lipid metabolism. This allowed the validation of hCA VA as an antiobesity target and started the research for new selective inhibitors towards this isoform as possible new antiobesity agents [36,37,38].

### 2.2. Thiophene Sulfonamides

**Figure 2:**
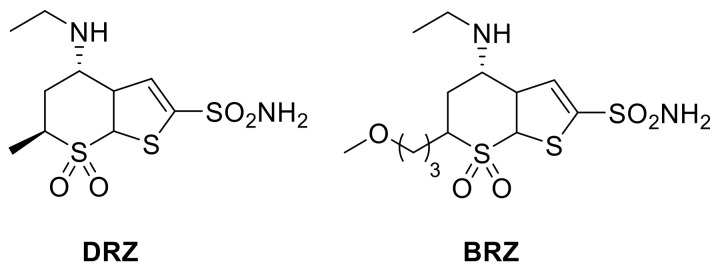
Structures of clinically approved dorzolamide (**DRZ**) and brinzolamide (**BRZ**).

Thiophene sulfonamide derivatives were studied as potential antiglaucoma drugs in the early 1990s by Shepard and colleagues [39,40]. In the same period, sulfonamide derivatives incorporating the bicyclic thieno-thiopyran ring were used as lead compounds for the development of the first clinically used, topically acting antiglaucoma sulfonamide, dorzolamide (**DRZ**) and, some years later, brinzolamide (**BRZ**, Figure 2).

**Scheme 6:**
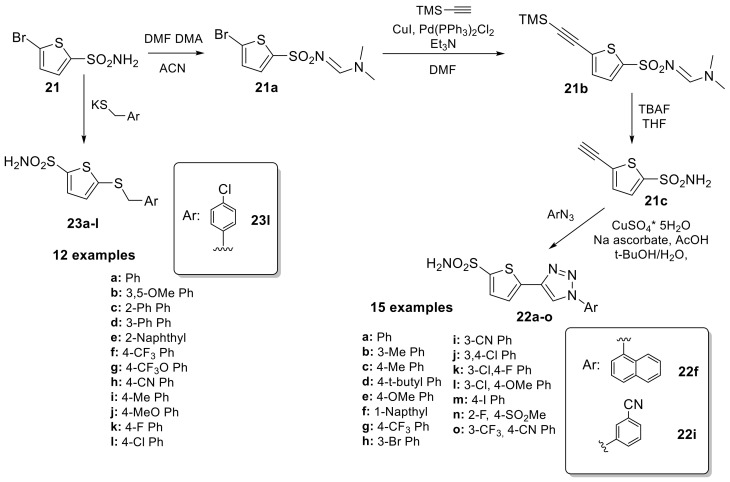
Synthesis of thiophene sulfonamides reported by Leitans et al.

Over the next 20 years, these sulfonamide derivatives were scarcely investigated as CAIs until 2013 where Leitans et al. [41] reported the synthesis of a series of 5-substituted-(1,2,3-triazol-4-yl)thiophene-2-sulfonamides, prepared by click chemistry from thiophene sulfonamide (**21**) as outlined in Scheme 6.

**Figure 3:**
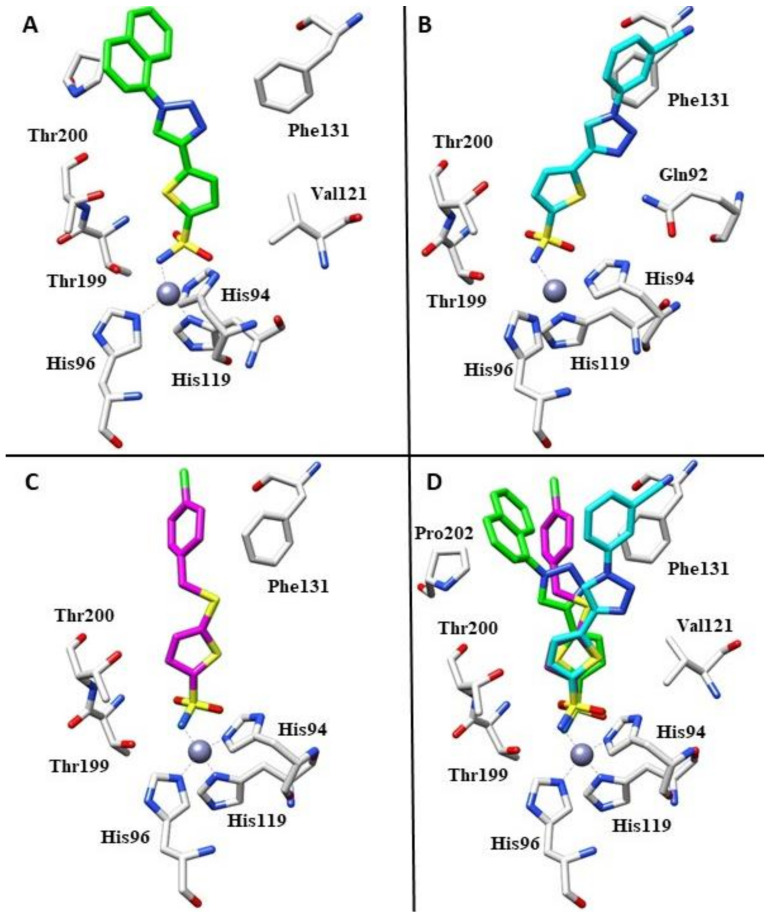
(**A**) Binding mode of compound **22f** within the active site of hCA II (PDB: 4BF1). (**B**) Binding mode of compound **22i** within the active site of hCA II (PDB: 4BF6). (**C**) Binding mode of compound **23l** within the active site of hCA II (PDB: 5MJN). (**D**). Superimposition of compounds **22f**, **22i** and **23l** in complex with hCA II.

The synthetic pathway involves the direct installation of an alkyne moiety via cross-coupling reaction only after protection of the sulfonamide group and a Cu(I) catalysed azide–alkyne cycloaddition was performed to obtain a series of fifteen 1,4-substituted 1,2,3-triazoles derivatives. Subsequently, the authors tried to rationalize the inhibition data by means of high resolution X-ray crystal structures for the adducts of 2**2f** and 2**2i** with hCA II (K_i_ 2.4 and 4.5 nM, respectively). In addition to the typical interaction of the deprotonated sulfonamide moiety with the zinc ion and the hydrogen bond between the NH of the sulfonamide and the OH of Thr199, commonly observed in most sulfonamide–hCA II complexes, compound **22f** does not participate in any other polar interactions. This is one of the few cases in which the positioning of the ligand within the hCA II active site was solely accomplished through van der Waals interactions (Figure 3A). 

Indeed, the naphthyl moiety was located well within a hydrophobic pocket of the protein defined by residues Phe131, Val135, Leu204 and Pro202. In contrast, compound **22i** showed different hydrophobic interactions with the side chains of residues Val121, Leu141, Phe131, and Leu198, resulting in a different orientation of the tail (Figure 3B). As observed by an overlapping of the two complexes (Figure 3D), only the sulfonamide groups of the two compounds are superimposable. The thiopene ring showed a different orientation, and aryl–triazolyl moieties of the two compounds adopted highly diverse orientations within the hCA II active site cavity. Compound **22f** was orientated towards the hydrophobic half of the hCA II active site, while compound **22i** was oriented towards the hydrophilic half, making them completely non-superimposable [41]. In 2017, the same authors reported a novel series of 5-thiophene-2-sulfonamide derivatives that introduced substituted-benzylsulfanyl moieties, as the SCH_2_ linker between the two aromatic fragments could lead to good flexibility of the inhibitor tail, increasing isoform selectivity [42]. From a general perspective, this series showed weak inhibition against hCA I (K_I_s in the range of 683–4250 nM), while the cytosolic enzyme hCA II was effectively inhibited in the nanomolar range. For this reason, the authors determined the crystal structure of the hCA II-**23l** complex using an X-ray study. Similarly to derivative **22f** mentioned above, compound **23l** did not exhibit other polar interactions except for the sulfonamide moiety. Its orientation was mainly determined through hydrophobic interactions with residues Val121, Phe131, Val135, Leu198, Thr200 and Pro202 (Figure 3C) [42].

**Figure 4:**
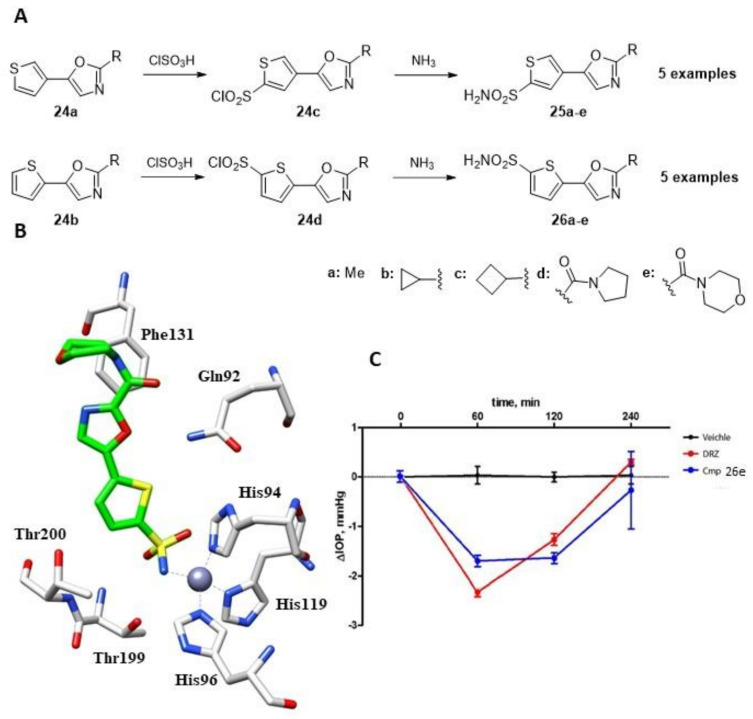
(**A**). Schematic synthesis of thiophene sulfonamides by Krasavin et al. (**B**). Binding mode of compound **26e** within the active site of hCA II (PDB: 5NEE). (**C**). The drop in intraocular pressure (ΔIOP, mmHg) versus time (min) in hypertonic saline-induced ocular hypertension in rabbits, treated with 50 µL of 1% solution of compounds **26e** using 2% dorzolamide (**DRZ**) solution as a reference drug and vehicle (saline).

Another interesting investigation of thiophene sulfonamide derivatives as CAIs was conducted by Krasavin et al. in 2015, who reported a small library of 10 derivatives with five compounds substituted at position 5 (**25a**–**e**) and the remaining five with substituents at position 4 (**26a**–**e**, Figure 4A) [43].

The 1,3- oxazole precursors (**24a**–**b**) underwent direct sulfochlorination, producing sulfonyl chlorides (**24c**–**d**) in high yields and as a single regioisomer. These were then converted into corresponding sulfonamide derivatives with aqueous ammonia (**25a**–**e** and **26a**–**e**). The 2,5-thiophene and 2,4-thiophene-linked derivatives showed an intriguing SAR pattern, generally exhibiting bias toward CA II inhibition. In the case of the 2,5-thiophene-linked compounds, small alkyl substitutions in the 1,3-oxazole ring (with the exception of cyclobutyl) resulted in weaker CA II inhibitors compared to the carboxamide substitutions in the same position. However, this pattern became scrambled for the 2,4-thiophene-linked counterparts, resulting in a loss of selectivity [43]. The authors then focused on derivative **26e** (Figure 4), which was studied using X-ray crystallography to determine its complex structure with hCA II [44]. The sulfonamide nitrogen was found to coordinate with the metal ion and engaged in a strong hydrogen bond with the OH of Thr199, as previously reported for compounds **22f, 22i** and **23l**. Additionally, one of the sulfonamide oxygen of the inhibitor formed a hydrogen bond with the amide nitrogen of Thr199. CA II is present in the ciliary body epithelium and promotes the formation of bicarbonate ions, which contribute to fluid transport and intraocular pressure (IOP). By inhibiting CA II, the formation of bicarbonate ions is reduced, resulting in decreased fluid transport and ultimately lower IOP.To investigate its capacity to reduce IOP, compound **26e** was tested in an ophthalmologic study. The derivative was administered directly into rabbit eyes, and IOP was measured for 4 h after administration, with the standard drug dorzolamide (**DRZ**) serving as a reference (Figure 4C). The IOP effect of compound **26e** was found to be slightly less effective than DRZ at 60 min after administration; however, its efficacy was better than the standard drug at later times, and its effect lasted longer than DRZ, whose effect was close to 0 at 4 h (Figure 4C) [44]. As a result of potent inhibition of hCA II by this series of compounds, applying the same strategy to other sulfonamide derivatives with similar inhibition profiles was thereafter considered [29,45].

### 2.3. Thiazole Sulfonamides

**Figure 5:**
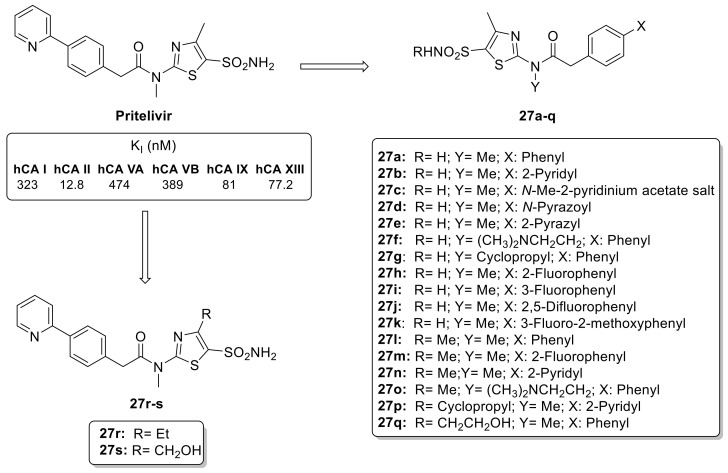
Structure of pritelivir and its derivatives **27a**–**s**.

The thiazole ring is a common scaffold in many natural compounds, such as thiamine (vitamin B1) and different types of antibiotics [46]. These properties make thiazoles a popular choice as building blocks in the synthesis of biologically active compounds, including anti-viral drugs [47]. Among them, Pritelivir (Figure 5), has recently been approved for clinical use for treating herpes simplex virus infections. Pritelivir incorporates a primary sulfonamide moiety not present in any other antiviral agent and potentially inhibits the different hCA isoforms [48]. Additionally, the use of elongated scaffolds in CAIs provides the opportunity to create compounds with high affinities for various hCA isoforms, often resulting in isoform-selective inhibitors. To develop pritelivir-like compounds, Carta et al. synthesized 5-sulfonamidethiazole substituted sulfonamides (**27a**–**s**) with chemical diversity, including six derivatives with a secondary sulfonamide moiety (SO_2_NHR) as highlighted in Figure 5 [49].

The library of synthesized primary and secondary thiazole sulfonamides was investigated against six hCA isoforms such as hCA I, hCA II, hCA VA, hCA VB, hCA IX and hCA XII. The data showed that the nature of the aromatic moiety in the tail of the molecule appears to be the most important factor influencing its activity against different isoforms. In particular, the 2-pyridyl moiety was found to be less effective than phenyl ring [49]. The replacement of one carbon atom with a nitrogen atom leads to important changes in the inhibitory activity, a phenomenon that has been previously documented in various classes of CAIs through kinetic and X-ray crystallographic studies [49]. There was no major difference in activity between primary and secondary sulfonamides for hCA I and II, as well as for the tumor-associated isoforms hCA IX and XII. Furthermore, the group in position 4 of the thiazole ring does not appear to have influence on the inhibitory properties of these sulfonamides derivatives [49]. 

**Scheme 7:**
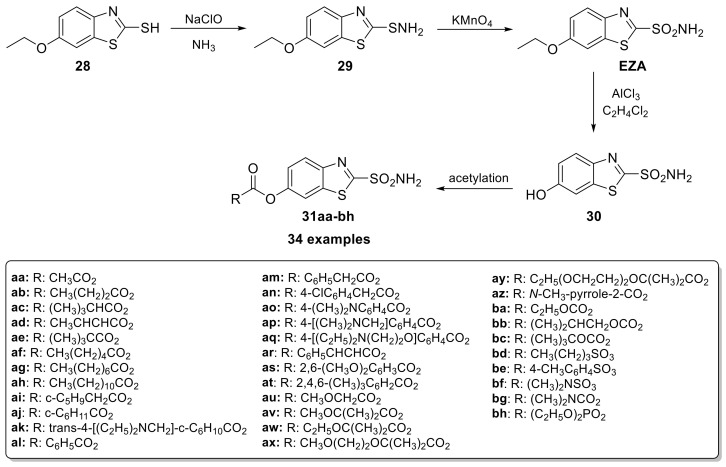
Schematic synthesis of O-acyl derivatives of 6-hydroxy benzothiazole-2-sulfonamide.

In 1982, ethoxzolamide (**EZA**) was approved by the FDA for the treatment of glaucoma, due to its ability to lower intraocular pressure (IOP) through CA inhibition [50]. Subsequently, Merck Sharp & Dohme Research Laboratories developed a series of O-acyl derivatives of 6-hydroxy benzothiazole-2-sulfonamide (Scheme 7) acting as a prodrug to enhance ocular absorption.

For its synthesis, the commercially available 6-ethoxybenzothiazole-2-thiol (**28**) is initially converted to the respective sulfenamide (**29**) and subsequently oxidized with potassium permanganate to **EZA**. This is followed by the ethyl ether cleavage with aluminum chloride and the final acetylation carried out at 0 °C in acetone or DMF using triethylamine as the base [51]. Among the synthesized derivatives (**31aa**–**bh**), the aliphatic acid esters showed the best activity; in particular, the compound with *tert*-butyl ester (R = C(CH_3_)_3_) was the best. However, the adverse ocular side effects evoked in animals (strong contact sensitizer) precluded its possible development as a clinical candidate [51].

**Scheme 8:**
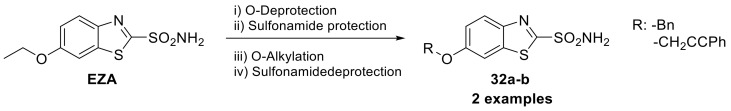
Schematic synthesis of ethoxzolamide (**EZA**) derivatives by Carta et al.

Therefore, to date, the only thiazole sulfonamide clinically used as CAI in the treatment of glaucoma is ethoxzolamide, [52]. In 2015, Carta et al. synthesized two ethers (**32a**–**b**) derived from ethoxzolamide (Scheme 8) as potent CAIs to employed as novel neuropathic pain relief [53].

Indeed, connections have been observed between the activity of CAs and pain reducing HCO_3_^-^-dependent depolarization via GABA_A_ receptors when the function of the neuron-specific potassium-chloride (KCl) cotransporter KCC2 is compromised [54,55]. The type of moiety bearing the phenol portion of the tail seems to be a crucial factor that impacts activity against different isoforms, as demonstrated in the case of hCA IX, where a phenyl moiety showed a 10-fold increase in potency compared to other reported derivatives [53]. 

### 2.4. 1,3,4-Thiadiazole- and 1,3,4-Thiadiazoline Sulfonamides

**Figure 6:**
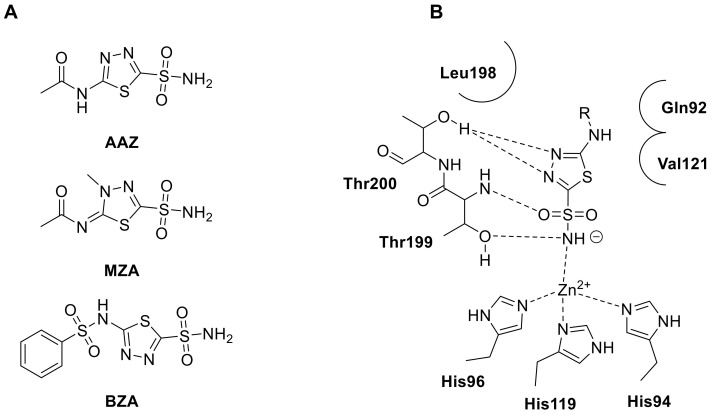
(**A**). Structures of clinically thiadiazole sulfonamide acetazolamide (**AAZ**), methazolamide (**MTZ**) and benzolamide (**BZA**). (**B**). Schematic representation of the binding mode of thiadiazole sulfonamide derivatives within hCA II active site.

The 1,3,4-thiadiazole scaffold, a five-membered heterocyclic ring composed of one sulfur and two nitrogen atoms, is a highly valued structure due to its unique chemical and biological properties and diverse range of biological activities [56]. Moreover, thiadiazole sulfonamides have been found to have low toxicity and minimal side effects, making them attractive candidates for further development as therapeutics agents [57,58,59,60]. Unlike thiophene, thiadiazole sulfonamides have been extensively studied as CAIs observing the thiadiazole ring participated to an additionally hydrogen bond interaction with the oxygen atom of Thr200 (Figure 6B) [52,61].

Acetazolamide (**AAZ**, Figure 6A) is a widely recognized example of the thiadiazole scaffold, which is composed of a five-membered heterocyclic ring containing one sulfur and two nitrogen atoms. **AAZ** has been used for decades as the first clinically approved CAI, and has been and is still used to treat a variety of medical conditions including edema, glaucoma, gastroduodenal ulcers, mountain sickness, epilepsy, tumors [16,17,18,19,20]. Following the success of **AAZ**, another thiadiazole sulphonamide (in fact a thiadizoline), methazolamide (**MZA**, Figure 6A), was discovered and has been used as an antiglaucoma drug for over 40 years [62,63]. The most recent thiadiazole sulfonamide to be introduced into clinical use is benzolamide (**BZA**, Figure 6A), which has unique physicochemical properties due to the presence of a highly acidic sulfonamide moiety (pKa of 3.4). This makes **BZA** much more polar than structurally related analogues **AAZ** and **MZA**, and as a result, it crosses biological membranes with more difficulty, restricting its uptake into the central and peripheral nervous systems [64]. For these reasons, the thiadiazole heterocyclic ring is still employed nowadays for development various analogues of **AAZ** to improve its therapeutic potential as well as to enhance its pharmacokinetic proprieties and address its limitations in obtaining novel drug candidates to employ in several diseases. 

#### 2.4.1. Thiadiazole Sulfonamides in Anticancer Terapy

**Scheme 9:**
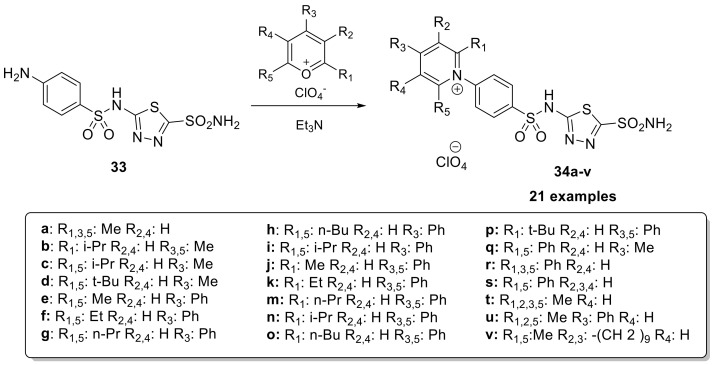
Schematic synthesis of positively charged thiadiazole sulfonamides **34a**–**v**.

In the last few years, many studies have validated the activity of CAI as an anticancer drug. Indeed, two transmembrane isoforms of CAs (CA IX and CA XII) are overexpressed in some tumors and are involved in many important tumor processes [65,66,67]. For these reasons, in 2003, a series of positively charged sulfonamides **34a**–**v** (Scheme 9) were developed to prevent the permeation of membranes and act selectively on the transmembrane CA isoforms overexpressed in the tumor [68].

For their synthesis, 5-((4-aminophenyl)sulfonamido)-1,3,4-thiadiazole-2-sulfonamide is reacted with pyrylium salts in anhydrous methanol in the presence of acetic anhydride as solvent and triethylamine as the base. The derivatives demonstrated significant inhibitory activity against all investigated isoforms. Specifically, for CA I, the inhibition constants ranged from 3–12 nM; for CA II, the range was 0.20–5.96 nM; and for CA IX the range was 3–45 nM. The high affinity of these derivatives for the tumor-associated isozyme, CA IX, combined with their membrane impermeability, makes them interesting candidates for selective inhibition of only the tumor-associated isoforms [68].

**Scheme 10:**
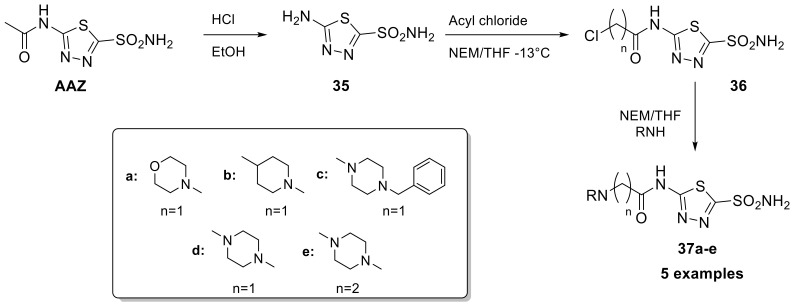
Schematic synthesis of acetazolamide derivatives **37a**–**c** by Turkmen et al. with the tail approach.

The following year, H. Turkmen, in collaboration with the Supuran research group, synthesized a series of derivatives also presenting the acetazolamide scaffold (Scheme 10) using the tail approach to increase the selectivity of the synthesized CA inhibitors towards the protumoral isoform CA IX [69]. The “tails approach” is an interesting strategy developed for the first time in 1999 for the synthesis of new isoform-selective CAIs. This consists of the application of a tail to a zinc-binding group in order to modulate the chemical-physical properties of the molecules and at the same time increase the interaction with the active site of the specific isoform of interest. The tail allows to interact with the residues of the middle and outer ridges of the binding pockets (areas with greater amino acid variability among the various isoforms) to improve the isoform specificity [70,71,72,73].

Subsequently, this approach was further developed in the “dual-tail” and “three-tails” approach to increase the possibility of interaction with specific residues of the target isoform and, at the same time, to have the possibility to interact simultaneously with both the polar and the lipophilic area of the binding pocket, presenting a hydrophobic and a hydrophilic tail [74,75,76,77,78].

**Figure 7:**
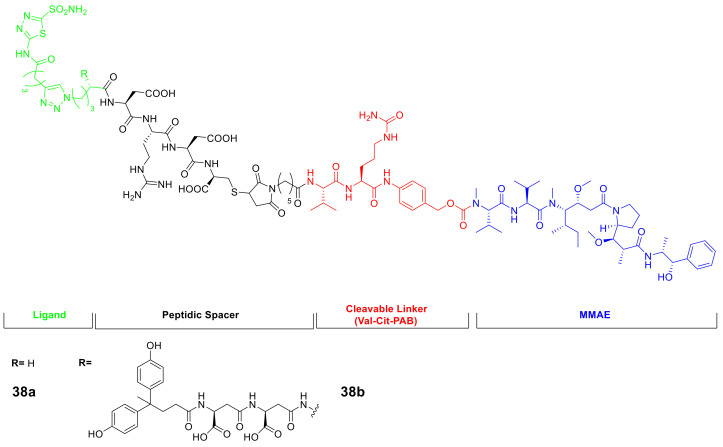
General structures of CA IX-targeting SMDCs incorporating the AAZ scaffold.

Another area of interest is the use of thiadiazole sulfonamides in the oncological field, where small molecule-drug conjugates (SMDCs) are being investigated at the preclinical and clinical levels. The hCA IX ligands have been considered as promising delivery vehicle for SMDC development. The Neri group has developed potent anti-tumor SMDCs based on **AAZ** conjugate with DM1, monomethyl auristatin E (MMAE), or the ultra-potent nemorubicin PNU-159682, which display improved tumor targeting of hCA IX through the use of novel AAZ derivatives and a cleavable peptide linker, as shown in Figure 7 [79,80].

**Scheme 11:**
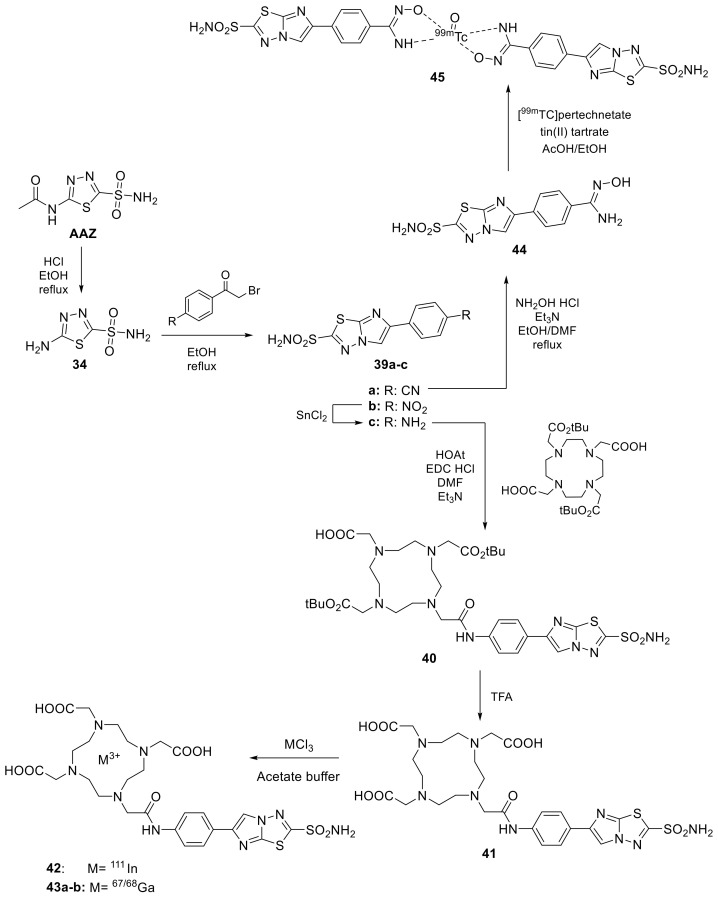
Schematic strategy to radiolabeling imidazothiadiazole sulfonamide synthesized by ONO group.

The results showed that the products had high levels of accumulation in the tumor mass (40%), present six hours after intravenous administration and a tumor-to-blood ratio of 80:1. Of the derivatives tested, compound **38b** (shown in Figure 7) was found to be well-tolerated in the in vivo model and demonstrated the most potent effect in inhibiting tumor growth. Moreover, **AAZ** was used as a starting point to obtain novel imidazothiadiazole sulfonamide as CAIs to employ in oncological field to due their high potency against the tumor-associated isoform hCA IX. In this context, the imidazothiadiazole sulfonamide backbone was employed for positron emission tomography (PET) imaging installing a metal chelator, such as 1,4,7,10-tetraazacyclododecane-1,4,7,10-tetraacetic acid (DOTA) and 1,4,7-triazacyclononane-1,4,7-triacetic acid (NOTA), which are widely utilized to conjugate with various radiometals for radiotheranostics with the increasing interest in precision medicine (Scheme 11). Considering the present scenario, the Ono group’ applying this CAIs to target hCA IX with different radiotracers for the single photon emission computed tomography (SPECT). In 2020, the authors prepared the ^111^In-labeling precursor with DOTA metal chelator (**42**) and radiolabelling this derivative with ^111^In by ligand substitution reaction using acetate buffer with a 76% of radiochemical yield and over 95% radiochemical purity [81]. 

The authors evaluated sulfonamide **42** against two tumor cell lines such as HT-29, where it showed abundant overexpression of hCA IX also in normoxic conditions compared to the other cell line MDA-MB-231. Subsequently, was performed an in vivo biodistribution assay using a tumor-bearing mice displayed an inhibitor uptake of 3.7–3.8% which was comparable to previously reported nuclear medicinal imaging probes targeting hCA IX [82,83]. Interestingly, radioactivity in the HT-29 tumor gradually increased with time and reached 8.71% injected dose/g at 24 h post injection and SPECT with 15 clearly and selectively visualized the HT-29 tumors in the living model mice [81]. The same authors, one year later, have replaced the radiometal ^111^In with ^67^Ga (**43a**) and ^68^Ga (**43b**) which has a much longer decay half-life (3.26 d) and can be utilized more easily than ^67^Ga (68 min) [84]. The obtained radiotracers **43a**–**b** (Scheme 11) showed similar radiochemical yield and purity than the previously one as well as the high radiolabelling in HT-29 cell line with respect to MDA-MB-231. Indeed, changing the radiometal (^111^In to ^67^Ga) did not affect its affinity. However, in vivo biodistribution assay was observed a less tumor uptake of ^67^Ga-derivative 16a compared to indium derivative (**42**) [84]. Finally, the Ono group’ reported an imidazothiadiazole sulfonamide derivative labelled with ^99m^Tc, the main isotope used in clinical practice, without DOTA instead the chelator agent hydroxamamide moiety (**45**, Scheme 11) [85]. This derivative anyway showed a lower tumor biodistribution compared mentioned above radiotracer with only 1.82% after 24h post injection. On the other hand, high radioactivity levels are observed in the stomach, suggesting the conversion of **45** to pertechnetate in vivo and low stability.

#### 2.4.2. Anti-Infective Thiadiazole Sulfonamides 

**Scheme 12:**
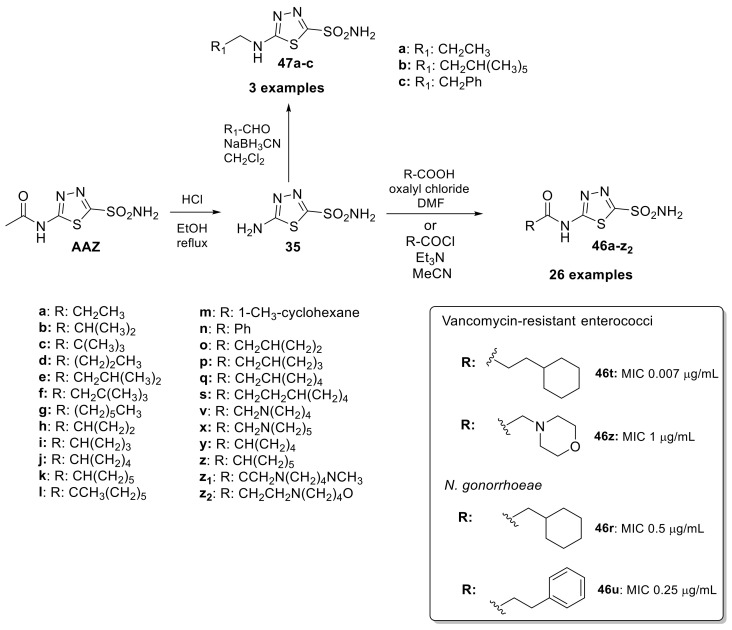
Schematic synthesis of acetazolamide-based derivatives by Flaherty group and MIC of derivatives **46**–**47** against Vancomycin-resistant enterococci and *N. gonorrhoeae*.

Drug repurposing has gained popularity as a fast and effective way to bring new treatments to patients and optimize the use of existing drugs. A recent example of this use was **AAZ** as a new class of anti-infective agents against bacteria such as enterococci and *Neisseria gonorrhoeae* strains [86,87]. Bacterial CAs play a crucial role in providing the necessary carbon dioxide and bicarbonate for microbial biosynthetic pathways. Therefore, inhibiting their activity can potentially impair the survival of pathogens. This scaffold, as mentioned above, represents a particularly attractive starting point for optimization because it has an overall safe profile, possesses good pharmacokinetic properties, rapid absorption and high renal clearance even if its low permeability limits its employment. Starting in 2020, the Flaherty group synthesized a library of 29 acetazolamide-based derivatives to optimize this scaffold [88]. The first synthetic step was cleaved the acetamide group of **AAZ** to provide the free amine (**35**) and undergone towards amide coupling (**46a**–**z_2_**) or reductive amination (**47a**–**c**) reactions to yield the final analogues as reported in Scheme 12.

Regarding the anti-Vancomycin-resistant enterococci (VRE), potency appeared to be enhanced by alkyl rings systems over aromatic pendent groups. In addition, lipophilic alkyl and phenyl substitutions improved potency and are likely to increase permeability through the intestinal lining compared to **AAZ**, making these analogs potential candidates for treating systemic VRE infections. Among the series, two potential leads shown to have a MIC of 0.007 μg/mL (**46t**) versus clinical strains of VRE and a MIC_90_ value of 0.03 μg/mL. On the other hand, derivatives **46z** had a measured MIC of 1 μg/mL and an MIC_90_ value of 4 μg/mL. From the data and experiments reported in this study, all CAIs were observed to be bacteriostatic conversely to azithromycin with bactericidal activity [88]. Regarding anti-infective activity against *N. gonorrhoeae* Strains, compounds **46r** and **46u** were investigated on a broad panel of 30 *N. gonorrhoeae* clinical isolates showed bacteriostatic properties with MIC of 0.5 μg/mL (13) and 0.25 μg/mL (**46u**) and shown to be nontoxic against two relevant human cell lines [89,90]. The data presented emphasizes the potential of this class of molecules to be included in the arsenal of effective therapeutics for the treatment of *N. gonorrhoeae* and Vancomycin-resistant enterococci.

#### 2.4.3. Thiadiazole-Sulfonamides in Central Nervous System Diseases

**Scheme 13:**
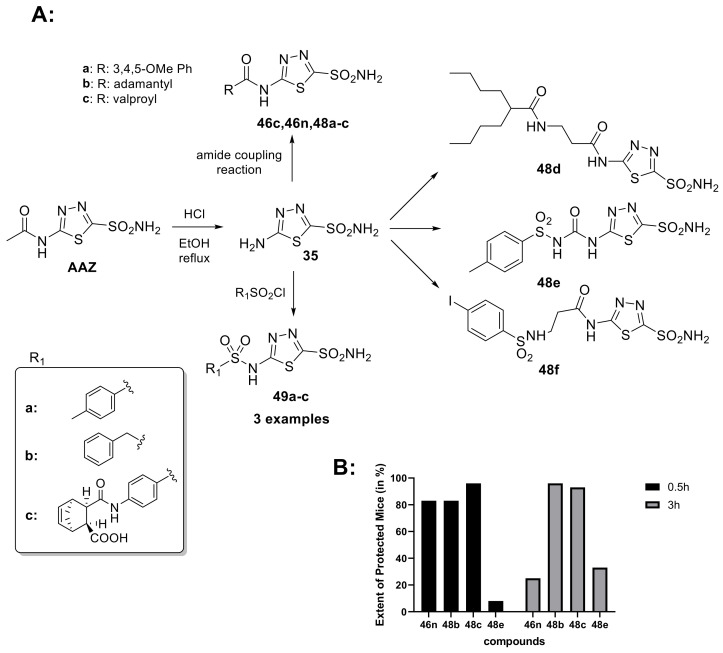
(**A**) Schematic synthesis of acetazolamide-based derivatives created by Masereel et al. to increase the lipophilic of **AAZ** scaffold. (**B**) Extent of protected mice (in %) against convulsions in the MES Test at 0.5 and 3 h following intraperitoneal injection of 30 or 10 mg/kg of CA inhibitor.

The anticonvulsant activity of CAIs has been known for a long time. Increasing levels of carbon dioxide in the brain is a potential treatment for seizures, and this can be achieved allopathically by using CAIs. In fact, in 1953, **AAZ** was approved by the FDA for the treatment of epilepsy; it is primarily used in combination therapy with other antiepileptic medications and also used in partial, myoclonic, absence and primary generalized tonic-clonic seizures in refractory epilepsy [20,91]. However, due to tolerance issues and side effects including metabolic acidosis and renal stones, its long-term use is limited [92]. Furthermore, it has been demonstrated that the increase in lipophilic on the **AAZ** scaffold leads to an improvement in the anticonvulsant activity, probably due to the better permeation in the brain [93]. For these reasons, in 2002, Masereel et al. developed a series of lipophilic derivatives of **AAZ** (Scheme 13) among which is also a derivative with valproic acid, another antiepileptic drug.

**Scheme 14:**
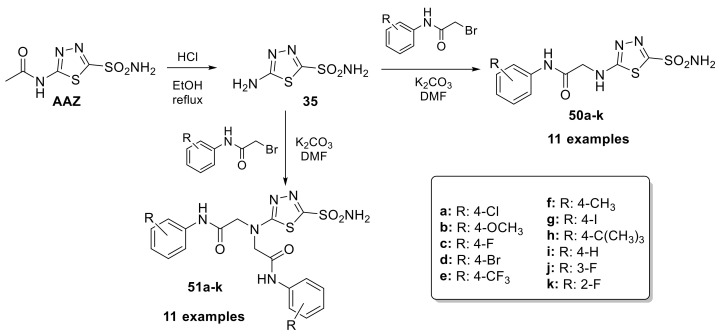
Schematic synthesis of acetazolamide-based derivatives by Zhang’s research group.

It has been observed that some derivatives, such as **46n**, **48b**, **48c** and **48e**, have shown promising anticonvulsant properties in vivo; furthermore, the valproyl derivative of acetazolamide (**48c**) showed the best results and exhibited very strong anticonvulsant properties in the MES test in mice. Therefore, these compounds can be considered interesting candidates for the development of new anticonvulsant drugs [94]. Given the widespread diffusion of CA in the brain and the importance of this enzyme for the central nervous system, in 2020, the J. Zhang research group synthesized a series of 5-[2-(*N*-(Substituted phenyl)acetamide)]amino-1,3,4-4 thiadiazole-2-sulfonamides derivatives (**50**–**51**) as selective hCA II inhibitors with neuroprotective effects (Scheme 14). 

In the same reaction, both mono (**50a**–**k**) and disubstituted (**51a**–**k**) compounds were obtained by adding the proper benzyl bromide to the solution of 5-amino-1,3,4-thiadiazole-2-sulfonamide and potassium carbonate in DMF at −20 °C [95]. The inhibitory activities of all compounds on human carbonic anhydrases I, II and IX were studied. The mono-substituted compounds showed better inhibition with significant selectivity for hCA II (the best IC_50_ = 16.7 nM). In addition, they showed neuroprotection on the PC12 cells from sodium nitroprusside (SNP)-induced damage [95].

#### 2.4.4. Thiadiazoles as Antiobesity Experimental Agents

**Figure 8:**
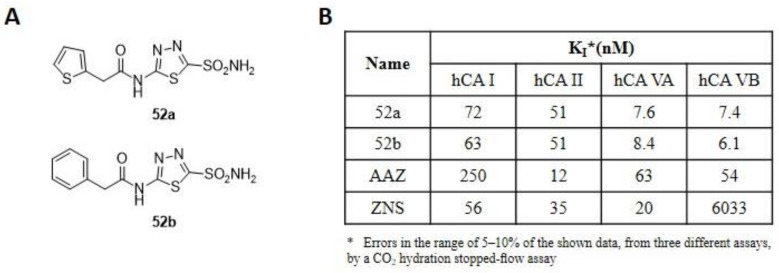
(**A**) Structure of thiadiazole derivatives **52a**–**b** synthesized by Supuran and Güzel research groups. (**B**) Inhibition data of human CA isozymes I, II (cytosolic), VA and VB (mitochondrial) with compounds **52a**–**b** and standard inhibitors (**AAZ** and **ZNS**).

The inhibition of mitochondrial CA isoforms (VA and VB) interferes with lipid metabolism, leading to a reduction in body weight [43,44,45]. For this reason, research has focused on the development of new selective CAIs towards these two isoforms as new anti-obesity agents. In this context, the Supuran research group, in collaboration with O. Güzel in 2009, developed a series of compounds, including two thiadiazole derivatives (**52a**–**b**, Figure 8A) to act selectively on mitochondrial hCAs.

**Figure 9:**
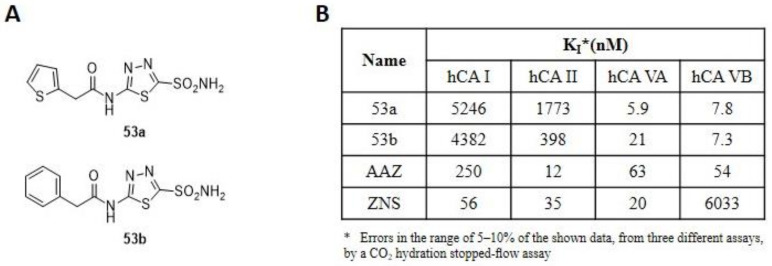
(**A**) Structure of thiadiazole derivatives incorporating *R*- and *S*-canphorsulfonyl moieties **53a-b**. (**B**) Inhibition data of human CA isozymes I, II (cytosolic), VA and VB (mitochondrial) with compounds **53a** (*R*), **53b** (*S*) and standard inhibitors (**AZA** and **ZNS**).

The compounds were obtained via SN reaction in anhydrous acetonitrile and were tested on the human cytosolic isoforms I and II (off target) and the mitochondrial VA and VB. The inhibition results (Figure 8B) showed a high activity towards mitochondrial isoforms compared to reference standards, with K_I_ in the low nanomolar range. Moreover, they showed good selectivity, with selectivity ratios for inhibition of mitochondrial over the cytosolic isoenzymes of >6 [96]. Two years later, a further series of compounds, incorporating *R*- and *S*-canphorsulfonyl moieties (**53a**–**b**) were synthesized to increase the selectivity towards both mitochondrial isoforms. Among these sulphonamides, **53a** and **53b** (Figure 9A), with thiadiazole scaffold, showed the best inhibitory profiles (Figure 9B).

The introduction of *R*- and *S*-canphorsulfonyl moieties increase the lipophilicity of the molecules to allow the permeation through the membranes, to reach the mitochondria where the CA VA and VB are located. The new derivatives showed a superior inhibitory capacity towards the mitochondrial isoforms compared to the cytosolic ones, with derivative **53a** that has a selectivity ratio of 889 for the inhibition of hCA VA over hCA I, and of 300.5 for the inhibition of hCA VA over hCA II, while the selectivity ratios for the inhibition of hCA VB over hCA I and of hCA VB over hCA II are 672 and 227, respectively. The chirality inversion of stereocenter in **53b** leads to an increase in activity on the off-target isoforms (I and II) and to a loss of inhibition towards the CA VA, while the activity on the VB isoform is almost unchanged [97]. To conclude, all the new synthesized derivatives have an excellent inhibitory profile and represent interesting leads for designing isoform-selective enzyme inhibitors targeting mitochondrial CAs involved in lipogenesis and obesity.

## 3. Conclusions

From the data presented in this review, it is clear that five-membered heterocyclic sulfonamides are a useful scaffold for the synthesis of new carbonic anhydrase inhibitors. Consequently, drug design studies for the design of potent and isoform-selective inhibitors have emerged over the last decades. However, only a rather limited number of such studies has been reported to date on oxygen and nitrogen heterocyclic sulfonamides, and their detailed pharmacological applications are unfortunately lacking. As one of the promising five-membered heterocyclic scaffold, the thiadiazole ring-bearing sulfonamide group, has achieved considerable success due to its pharmacological safety profile such as the clinically approved drugs acetazolamide or metazolamide. Although we report only a small fraction of thiadiazole derivatives, most of these compounds have demonstrated favourable biological and pharmacological properties, such as antibacterial and antitumor activities among others. Overall, the further development of CA inhibitors with five-membered heterocyclic rings is a promising area of research that could lead to the discovery of new drugs for the treatment of various diseases. However, more research is needed to fully realize the potential of these compounds and to bring them to the clinic.

## Data Availability

No data are available for this review article.
